# Impact of various types of heat processing on the energy and nutritional values of goose breast meat

**DOI:** 10.1016/j.psj.2021.101473

**Published:** 2021-09-06

**Authors:** Zuzanna Goluch, Król Barbara, Gabriela Haraf, Janina Wołoszyn, Andrzej Okruszek, Monika Wereńska

**Affiliations:** ⁎ Department of Food Technology and Nutrition, Wroclaw University of Economics and Business, Wroclaw 53-345, Poland; † Department of Animal Nutrition and Feed Science, Wroclaw University of Environmental and Life Sciences, Wroclaw 51-631, Poland

**Keywords:** Geese, breast muscle, heat treatment, energy value, nutritional value

## Abstract

The purpose of this study was to examine the impact of various types of heat processing used by consumers (water bath cooking **WBC**, oven convection roasting **OCR**, grilling **G**, pan frying **PF**) on the energy and the nutritional value of goose breast meat (with and without skin). The material used in the study comprised 72 breast muscles cut from carcasses of 17-wk-old White Koluda geese. The energy value (MJ), the chemical composition (water, fat, protein, ash) and mineral composition (phosphorus P, sodium Na, calcium Ca, potassium K, magnesium Mg, iron Fe, zinc Zn, cooper Cu, manganese Mn) were determined in both raw and thermally processed muscles. It has been concluded that various methods of heat processing have a significant impact on the energy and nutritional values of meat. From a dietary point of view, the most beneficial was OCR meat without skin, and WBC, OCR, PF meat with skin as well, since it had the lowest energy value as well as content and retention of fat, phosphorus, and sodium. However, as for the content of the other minerals and their retention, WBC seems to be the optimal form of heat treatment of skinless muscles. 100 g of such meat provides 3.1; 33.7; 145; 180 and 9% Nutrient Reference Values-Requirements (**NRVs-R**) for Ca, Mg, Fe, Cu, and Mn respectively in a diet of an adult person. As for meat with skin, the optimal method of heat processing to retain minerals is grilling. 100 g of meat processed in this way provides 3.9; 39.7; 125.7; 175; 6 and 12.7% NRVs-R of Ca, Mg, Fe, Cu, and Mn. It follows from the above information that goose breast meat, as analyzed here, cannot be considered as a source of calcium since it provides less than 4% of NRVs-R. The results of the study will be useful for the consumers’ nutritional choices. The geese breast meat, depending on the heat processing used and the content of skin, may be a valuable component of a varied diet, providing nutrients and minerals.

## INTRODUCTION

The sharp increase in civilization diseases can be observed in the global population (Kopp, 2019). They result from genetic factors, which cannot be modified, and diet-related factors, which can be modified (Franzago et al., 2020). Therefore, a varied and balanced diet, adjusted to one's age, sex, physiological condition, and the frequency and intensity of physical activity, is an important element of dietary prevention and therapy of civilization diseases. Contemporary consumers are aware of how the amount, quality, and safety of food affect one's health (Cena and Calder, 2020). The relevance of poultry meat for human diet also has been recognized by the UN Food and Agricultural Organization (Ravindran, 2013), which considers this widely available, relatively inexpensive food to be particularly useful in developing countries, where it can help to meet shortfalls in essential nutrients. The meat of waterfowl has specific nutritional and culinary value, but due to its high prices, it is less popular than the meat of gallinaceous poultry (Nowicka, 2018; Boz et al., 2019). To source and sell this meat commercially in the global market, domestic geese (of various breeds and types) and wild geese are used (Geldenhuys et al., 2016; Kozák, 2019). The largest producers of goose livestock in the world are China and Egypt, while in Europe – Poland and Hungary (FAOSTAT, 2021). In Poland, 95% of the production of goose meat involves White Koluda geese, which has been considered a goose breed since 2012 (Lewko et al., 2017).

Literature concerning slaughter geese focuses mainly on the influence of breed, keeping, feeding, sex, age, and the type of muscle on carcass traits and meat quality (Damaziak et al., 2016; Geldenhuys et al., 2016; Uhlířová et al., 2018; Wołoszyn et al., 2020a; Gumułka and Połtowicz, 2020; Haraf et al., 2021). However, in the research of the last 30 yr, there have been few studies on the content of minerals in goose meat. Mineral elements are nutrients that play a crucial role in the everyday diet of a human being, as they perform regulatory functions in the biochemical processes in the cells and tissues of a human body. Moreover, they influence the biological activity of the enzymes included in the meat, its osmotic qualities, its pH, and its sensory attributes. In the literature, one can most often find works on the content of heavy metals in the muscles and/or liver of goose due to the risk of health hazards to the consumer (Cieślik et al., 2011; Horak et al., 2014; Geldenhuys et al., 2015).

There were 4 reasons for undertaking this research. The first reason is the fact that globally, there is an increase in the use of mineral fertilizers in agricultural production, as has been shown in FAO reports (FAO, 2019) and the reports of the European Commission (European Commission, 2019). The use of fertilizer on a global level is rising on an annual basis by around 2% for P and K. Nitrogen (N), P, and K are the main components of fertilizers, but compound fertilizers often contain secondary macronutrients, such as calcium, sulfur, and magnesium. In many compounds, micronutrients are also added to fertilizers (i.e., Cu, Fe, Mn, B), which lead to their variable presence in grains, green whole-crop cereals, meadow green forages and silages, and root plants used to feed geese. Moreover, in large-scale animal production, goose feed is supplemented with Ca, Na, Fe, Mn, Cu, Zn, J (iodine), Co (cobalt), and Se (selenium) in the premixture, which may affect their content in the produced meat (López-Alonso, 2012).

The second reason is the fact that, throughout the years, the requirements concerning energy and nutritional value of the feed used in geese fattening have changed. In 1984, a higher supply of Ca and P in goose fattening up to the 4th wk was recommended than in 1994 (NCR, 1984, 1994; Applegate and Angel, 2014). Currently, The Committee of Nutrient Requirements of Poultry is working on the 10th edition of the feeding requirements for poultry. Third, geese must have access to adequate for their age, stage of production, and weather conditions amount of water (Berger, 2006). Water is also a source of minerals in the diet of birds, and its mineral composition is also variable. For the reasons outlined above, research published earlier that concerns mineral content in goose meat may not reflect the current knowledge on the actual mineral content in this meat.

Fourth, various heat processing methods used for the meat by consumers (depending upon environmental, economic, and cultural traditions) do not only ensure health, safety, sensory qualities, and digestibility but may also modify the energy and nutritional values of the meat, including the retention of minerals (Sobral et al., 2018; Yong et al., 2019).

What is more, consumers have been advised that to improve their blood lipid profile and/or to reduce their weight, they should remove the skin and subcutaneous fat from their meat (as it is the source of fat and cholesterol). However, poultry skin contains not only sulfur amino acids, collagen, elastin, and vitamins that dissolve in fats, but also minerals (Soriano-Santos, 2010; Marangoni et al., 2015; Stangierski and Leśnierowski, 2015).

Therefore, the purpose of this study was to examine the effects of different methods of heat processing (imitating the behavior of contemporary consumers) on the energy and nutritional value of geese breast muscles (with and without skin) and to find the optimal heat processing method which allows retaining these values.

## MATERIAL AND METHODS

### Meat Samples

The experimental material involves breast (*Pectoralis major*) muscles cut from carcasses of 17-wk old females of White Koluda geese, after fattening named “Polish oat geese.” The geese were fed and maintained in a specific way, that is, kept in open-air runs and pastures. They were reared and fattened up to 17th wk of age according to the standard of Polish fattening technology of White Kołuda geese. The birds were fed a complete concentrated diet. The concentrate mixtures were formulated according to Nutritional Recommendations and Nutritional Value of Feeds for Poultry and met geese requirements for nutrients in all stages of fattening. The starter mixture (from first to the fourth wk) was characterized by 19% protein and 11.9 MJ/kg, the grower mixture (from fifth to the eighth wk) contained 17% protein and 11.7 MJ/kg, and the finisher mixture (from ninth to 13th wk) included 14% protein and 11.7 MJ/kg. The main components of the commercial mixture were ground wheat, barley, triticale, oat, grass meal, and protein concentrate in varying proportions. From 14th to 17th wk of age, the birds were fattened freely with oat grain and grass meal (Biesiada-Drzazga et al., 2011; Buzała et al., 2014; Wojciechowski, 2016; Wołoszyn et al., 2020a). The oat grain intake during the last 3 wk of the fattening period was 500 g/d. Throughout rearing, the goose diets were supplemented with commercial 2.5% premixture included minerals, vitamins, and essential amino acids for fattening geese. The premixture was applied to the birds’ diets in accordance with manufacturer recommendations. The sources of minerals in premixture were inorganic compounds commonly used in the feed industry. The nutritional value and mineral composition of concentrate mixtures are presented in Table 1.

**Table 1:** Nutritional value and mineral composition* of concentrate mixtures for geese in various stages of fattening.

Item	Unit	Concentrate mixture			
		Starter	Grower	Finisher	Oat grain/Grass meal
Nutrient					
Dry matter	%	87.8	87.7	87.7	87.4
Crude protein	%	19.02	17.04	14.05	11.93
Crude fiber	%	3.91	4.92	8.65	8.96
ME	MJ/kg	11.9	11.7	11.7	12.84
Lysine	%	0.98	0.84	0.77	0.48
Methionine	%	0.47	0.43	0.35	0.19
Mineral composition					
Ca	g/kg	9.57	9.24	6.47	6.54
Pabs (absorbable phosphorus)	g/kg	4.30	5.23	5.16	1.23
K	g/kg	7.12	7.53	6.87	4.58
Na	g/kg	1.67	1.74	1.64	1.89
Cl	g/kg	1.41	1.32	1.31	1.04
Mn	mg/kg	108.5	113.4	113.7	118.3
Zn	mg/kg	98.64	99.71	99.35	89.37
Fe	mg/kg	126.1	125.6	124.2	104.7
Cu	mg/kg	20.96	21.42	21.33	19.54

⁎ 2.5% remixture provided to concentrate mixtures and oat grain: Ca – 5.8 g, Pabs – 2.8 g., Na – 1.45 g, Mn – 80 mg, Zu – 70 mg, Fe – 45 mg, Cu – 15 mg, I – 1.20 mg, Se – 0.35 mg, Lys – 0.83 g, Met – 1.0 g, vit. A – 7000 IU, vit. D3 – 2125 IU, vit. E – 25 IU, vit. B1 – 3 mg, vit. B2 – 5 IU, biotin – 150 mg, vit. B6 – 5 mg, vit. B12 – 35 mg, vit. K – 3 mg, niacin – 40 mg, folic acid – 4 mg, Ca pantothenate – 13 mg, choline – 225 mg.

The geese coming from the same commercial farm were slaughtered industrially. The eviscerated carcasses were placed into a 2°C to 4°C cooler for 24 h and then the breast muscles were cut out of the carcasses. Meat samples (N = 72, 24 raw and 48 cooked breast muscles) were standardized for thickness and weight (average weight for breast muscles with skin and subcutaneous fat ≈ 472 g, without skin ≈ 328 g).

### Heat Treatments

The methods of heat processing selected in our study are water bath cooking, grilling, pan frying (without fat or oil), and oven convection roasting, as these are methods commonly used in the domestic preparation of poultry meat. No salt (NaCl), spice, or any food additives were used in the experiment. A total of 48 breast muscles were used in each kind of heat processing (6 samples with skin and 6 samples without skin were investigated). After heat processing, muscles were allowed to cool to room temperature (for approximately 2 h). Each raw and cooked breast muscle was chopped separately (mesh diameter of 3 mm) in an electric bowl chopper (model MM/1000/887 Zelmer, Rzeszów, Poland).

#### Oven Convection Roasting (OCR)

Each breast muscle (wrapped in aluminum foil) was roasted in the forced-air convection oven (model EB7551B Fusion, Amica Ltd., Wronki, Poland) until the internal temperature of each sample was 75°C (25 min). Before roasting the oven was preheated at T = 200°C. The temperature in the center of each muscle was monitored using Teflon-coated thermocouples (Type T, Omega Engineering Inc., Stamford, CT) attached to a Doric multichannel data logger (VAS Engineering Inc., San Diego, CA).

#### Pan Frying (PF)

Pan frying was performed using an electric pan (model 48155, Unold AG, Hockenheim, Germany). The muscles were fried on a preheated pan (at 160°C) and turned when they reached an internal temperature of 40°C. Processing was completed after 15 min when the temperature in the geometric center of each sample (monitored with a hand-held thermometer) was 75°C.

#### Water Bath Cooking (WBC)

The breast muscles were placed (each separately) in thin plastic bags. Next, they were immersed in a water bath at a temperature of 90°C (model SW 22, Julabo GmbH, Seelbach, Germany). Samples were boiled for 30 min to reach 75°C inside (monitored with a hand-held thermometer).

#### Grilling (G)

Whole muscles were placed between 2 heating plates (heating on the bottom and top plates) of an electric grill (model PD 2020R, Red Fox, Warszawa, Poland), preheated to 200°C. The samples were grilled for 25 min until a final internal temperature of 75°C was reached (monitored with Teflon-coated thermocouples).

### Chemical Analysis

The basic chemical content of raw meat (12 muscles with skin and 12 muscles without skin) and the meat subjected to heat processing (6 muscles with skin and 6 muscles without skin for each type of heat processing) were analyzed with the use of reference methods, following the official analytical methods of the EN ISO 9831:2004 and AOAC (2016). The following energy and nutrient contents were determined: gross energy, measured in calories with the use of calorimeter KL-10 (PRECYZJA-BIT PPHU Sp. z o.o. Bydgoszcz, Poland); moisture content (%), measured by the oven-drying of 2 g samples at 102°C for 12 h to a constant weight in a SUP-4M laboratory dryer (Wawa-Med, Warsaw, Poland) (950.46B, p. 39.1.02); total nitrogen, measured with the Kjeldahl method converted (a conversion factor 6.25) into an amount of crude protein (%) on the Kjeltec 2300 Foss Tecator distiller (Häganäs, Sweden) (992.15, p. 39.1.16); and crude fat content (%), measured by petroleum ether extraction using a Büchi Extraction System B-811 (BÜCHI, Switzerland) (960.39 (a), p. 39.1.05). The ash (total mineral content %) was determined by incineration at 550°C for 10 h in a FCE 7SHM muffle furnace Czylok (Jastrzębie Zdrój, Poland) (920.153, p.39.1.09).

### Mineral Analysis

The minced meat was frozen for 12 h at a temperature of -18°C, and freeze-dried for about 48 to 72 h (underpressure, temperature of -55°C) depending on the size of the sample, in the freeze-dry apparatus from Modulyo (Edwards, Great Britain) to achieve a constant mass of the sample. Next, the freeze-dried samples were ground in a laboratory grinder WŻ-1 (Zakład Badawczy Przemysłu Piekarskiego Sp. z o.o., Poland).

The samples (0.3g for Ca, Mg, K, Na and 1.0g for Cu, Mn, Zn, Fe) of the freeze-dried goose meat were wet digested with 7 mL of HNO_3_-H_2_O_2_ mixture (2:3, v/v) using a MarsXpress microwave oven (MARS 6 Microwave Reaction System CEM Corporation, Matthews, NC). The wet mineralization program was the following: first (10 min), the temperature was increased to 190°C; secondly (7 min), the temperature was kept at a level of 195°C. Digested samples were placed in polypropylene tubes and diluted to 50 mL with ultrapure water. A blank digest was made in the same way. The concentration of macro- (K, Na, Ca, Mg) and microelements (Zn, Fe, Mn, Cu) was determined using the flame atomic absorption spectrometry (FAAS, air-acetylene flame) using a AA 240FS SIPS20 spectrometer (Varian, Mulgrave, Australia), according to the procedures of AOAC (2005). The content of macro- and microelements in the samples was expressed in milligram per 100 g of dry mass (**DM**).

The P content in goose meat was determined after previous mineralization samples with HNO_3_ (65%) and HClO_4_ acid in close microwave mineralizer MarsX9 (MARS 6 Microwave Reaction System CEM corporation Mattehews). It was analyzed spectrophotometrically by the ammonium vanadomolybdate method using a Specol 11 (Carl Zeiss, Jena, RFN) at a wavelength of 470 nm (AOAC, 2005). The content of P was expressed in milligrams per 100 g DM.

To determine the mineral composition of feedstuff and meat samples as well as the accuracy of analytical methods, wheat flour was used as the standard reference material (SRM 1567b, National Institute of Standards & Technology, USA https://www.nist.gov/srm). The determined concentrations (mg x kg^−1^) of Ca, P, Mg, K, Na were 198 ± 20.4, 1198 ± 127, 356 ± 5.3, 1304 ± 158, 6.74 ± 0.80 (n = 3), respectively, with all macroelements recovery ranged from 89.4% to 103.4%. Fe, Zn, Mn, Cu concentrations were also tested using SRM 1567b with trace minerals recovery ranged from 97.0 to 104.2% of certified values declared by SRM manufacturer.

### Determination of Retention Factors

The percentage of nutrient retention after heat processing was calculated by using the following equation (Bognár and Piekarski, 2000):

$$\%\;\text{Retention}=\;\frac{\;\text{Nutrient}\;\text{content}/100\;g\;\text{of}\;\text{meat}\;\text{after}\;\text{heat}\;\text{processing}\;}{\text{Nutrient}\;\text{content}/100\;g\;\text{of}\;\text{raw}\;\text{meat}}\times \frac{\text{meat}\;\text{weight}\;\left(g\right)\;\text{after}\;\text{heat}\;\text{processing}}{\text{meat}\;\text{weight}\;\left(g\right)\;\text{before}\;\text{heat}\;\text{processing}}\times \;100$$

### Statistical Analysis

Obtained results were examined for normality of distribution with Shapiro-Wilk Test and variation of homogeneity with Laven's test. The findings were log-transformed to attain or approach a normal distribution, and subsequently, a 2-way analysis of variance (ANOVA) was performed. Statistical significance of differences between the averages of the groups was calculated using Tukey's multiple comparisons test, on the level of significance *P* ≤ 0.05 and *P* ≤ 0.01, with the use of Statistica13.1 software. The tables show arithmetic means (x) and standard errors of the mean (SEM). All data are reported as means (±SEM) of 2 parallel measurements.

## RESULTS AND DISCUSSION

### Proximate Composition

Generally speaking, the meat of waterfowl is characterized by a higher energy value than that of gallinaceous poultry, due to its higher lipid content. Contemporary consumers pay attention to the energy value of their food, including meat (Pathare and Roskilly, 2016). The purpose of cooking meat is to reduce the microbiological hazards (extend the shelf life and inactivate antinutrient enzymes) and improve the digestibility and bioavailability of nutrients.

According to the data collected by 2020, the energy value of domestic geese skinless meat is 0.67, and with skin - 1.55 MJ/100 g DM. The Polish Food Composition Tables (Kunachowicz et al., 2017) written at the National Food and Nutrition Institute, only provide the energy value for raw muscles of the whole goose carcass (1.416 MJ/100 g DM), with no regard for the type of muscle and presence of skin. The energy values of raw and heat-processed breast muscles of geese that were measured are presented in Table 2. The lowest average energy value (0.94 MJ/100 g DM) (*P* ≤ 0.01) was found in raw breast muscles, both with and without skin (0.71 and 1.17 MJ/100 g DM), in comparison to heat-processed muscles. A lower energy value (0.56 MJ/100 g DM) for skinless breast muscles of wild Canada Goose (*Branta canadensis*) was provided based on 2020. The heat processing affects the rise in total energy value of muscles: G>PF>OCR>WBC, in comparison to raw muscles (*P* ≤ 0.01). Moreover, it was found that muscles with skin had a higher energy value (*P* ≤ 0.01) than skinless muscles (1.23 vs. 1.02 MJ/100 DM), which is related to higher content of intramuscular fat (13.9 vs. 5.18%). An interaction was also noted between the type of muscle (with or without skin) and the type of heat processing applied. Similarly, Belinsky and Kuhnlein (2000) found that the type of heat processing (oven-roasting OR, boiling BO, fire-roasting FR) affected the energy value of breast muscles with skin of Canada Goose: OR>BO>FR (1.22 >1.05>0.93 MJ/100 g DM respectively). On the other hand, the data in 2020 show that the energy value of roasted meat from goose carcass with and without skin was higher (0.99 and 1.18 MJ/100 g DM respectively) than the value we found in breast muscles. The difference may be explained by the presence of other elements in a goose carcass, such as leg muscles, which are characterized by higher contents of intramuscular fat.

**Table 2:** Energy value and basic chemical composition of raw and heat-treated White Kołuda goose muscles (MEAN, SEM).

Item	Meat	Raw	Thermal treatment					SEM	Level of significance		
			Water bath cooking (WBC)	Grilled (G)	Oven convection roasting (OCR)	Pan fried (PF)	Total				
									Meat (M)	Thermal treatment (MT)	M x MT
Gross energy (MJ/100 g)	Without skin	0.71^B^	1.71^A^	1.24^A^	1.13^A^	1.13^A^	1.02^Y^	0.06	0.000	0.000	0.001
	With skin	1.17	1.18	1.25	1.28	1.30	1.23^X^	0.03			
	Total	0.94^B^	1.18^A^	1.25 A	1.21 A	1.22 A	1.12	0.04			
	SEM	0.08	0.03	0.04	0.04	0.04					
Moisture (%)	Without skin	72.7^A^	58.4^B^	54.4^B^	58.1^B^	58.4^B^	62.5^X^	1.81	0.000	0.000	0.000
	With skin	61.7^a^	58.1	56.3	55.0^b^	56.7	58.3^Y^	0.84			
	Total	67.2^A^	58.3^B^	55.4^B^	55.6^B^	57.6^B^	60.4	1.05			
	SEM	1.79	0.27	1.20	0.85	0.64					
Protein (%)	Without skin	22.3^B^	33.2^A^^b^	36.4^A^^a^	33.8^A^	33.3^A^^b^	30.2^X^	1.40	0.000	0.000	0.016
	With skin	17.2^B^	29.9^A^	30.8^A^	30.5^A^	31.0^A^	26.1^Y^	1.57			
	Total	19.7^B^	31.6^A^	33.6^A^	32.2^A^	32.2^A^	28.1	1.09			
	SEM	0.85	0.87	1.43	0.78	0.67					
Fat (%)	Without skin	3.3^B^^b^	6.3^a^	6.7^A^	5.5	6.0^a^	5.18^Y^	0.41	0.000	0.726	0.002
	With skin	19.9^A^^a^	10.1^B^	10.2^B^	12.4^B^^b^	10.5^B^	13.9^X^	1.25			
	Total	11.6	8.2	8.57	8.9	8.3	9.5	0.98			
	SEM	2.68	0.87	1.10	1.69	1.04					
Ash (%)	Without skin	1.23^B^^d^	1.28^B^	1.74^A^^a^	1.38^B^^b^	1.54^b^	1.40^X^	0.05	0.000	0.000	0.000
	With skin	0.97^B^	1.19^A^^b^^C^	1.47^AD^	1.41^A^^a^^D^	1.48^AD^	1.25^Y^	0.06			
	Total	1.10^D^	1.24^C^	1.61^A^	1.40^B^	1.51^A^^B^	1.33	0.04			
	SEM	0.04	0.04	0.07	0.03	0.03					
Protein retention (%)	Without skin	-	93.7	95.5	91.8	94.7	93.9	1.10	0.302	0.607	0.704
	With skin	-	94.8	87.5	86.7	94.8	90.9	2.75			
	Total	-	94.3	91.5	89.3	94.7	92.4	1.48			
	SEM	-	3.01	3.32	2.35	3.28					
Fat retention (%)	Without skin	-	103.2	103.0	87.2	97.8	97.8^X^	6.72	0.001	0.976	0.684
	With skin	-	30.4	28.3	32.9	30.5	30.5^Y^	2.09			
	Total	-	66.8	65.6	60.1	64.2	64.2	7.81			
	SEM	-	17.2	18.4	15.2	15.8					
Ash retention (%)	Without skin	-	65.7	82.4	67.8	79.1	73.7	2.71	0.156	0.003	0.448
	With skin	-	71.0	78.7	75.2	84.8	77.4	2.08			
	Total	-	68.4^b^^B^	80.6^a^	71.5	81.9^A^	76.7	1.71			
	SEM	-	3.42	2.49	2.25	2.08					

Means within a row followed by different superscript letters differ significantly.

A,B,C,D *P* ≤ 0.01.

a,b *P* ≤ 0.05. Means within a row followed by different superscript letters differ significantly.

X,Y *P* ≤ 0.01. Means within a column followed by different superscript letters differ significantly. (n = 12 raw breast muscles with skin and n = 12 without skin; n = 6 breast muscles with skin and n = 6 without skin for each kind of heat treatment).

The effect of heat treatment on the increase in the energy value of meat can be explained by higher dry matter content in meat resulting from cooking loss (Wołoszyn et al., 2020b).

#### Moisture

The moisture content in raw breast muscles with skin of White Kołuda geese (61.7%) was lower than that found by Gumułka and Połtowicz (2020) in the muscles of 10-wk-old geese of the same breed (75.02%), and that found by Oz and Celik (2015) in the muscles of Turkish geese (66.38%). On the other hand, lower moisture content (57.93%) in raw breast muscles with skin of White Kołuda geese was reported by Damaziak et al. (2016).

Both the type of muscle (with and without skin) and the type of heat processing impacted (*P* ≤ 0.01) the moisture content in breast muscles of geese. The skinless muscles were characterized by higher content of moisture than those with skin (62.5 vs. 58.3%). The interaction between types of muscles (with and without skin) and the type of heat processing was also significant (*P* ≤ 0.01). The content of moisture in the raw muscles without skin (72.7%), was significantly (*P* ≤ 0.01) higher than in heat-treated samples. However, the moisture content was lower (*P* ≤ 0.05) in muscles with skin OCR, in comparison to raw muscles (55.0 vs. 61.7%). Similarly, Belinsky and Kuhnlein (2000) found that heat processing influences the moisture content in breast muscles with skin of Canada Goose: FR>B>OR (56.1>51.4>50.6% respectively), while Oz and Celik (2015) have found no significant impact of 7 types of heat processing (boiling, grilling, pan frying without fat or oil, pan frying with oil, deep-fat frying, oven roasting, microwave cooking) on the fluctuations in moisture content of Turkish geese breast muscles.

Thermal processing leads to release and evaporation of free water from the muscles. Consequently, the decrease in humidity on the surface of the product is observed. It gives rise to meat drying and reduces water activity. The reduction in water content in heat-treated muscles results from water loss during cooking, baking, grilling, and pan-frying due to evaporation and dripping. Cooking leads to structural changes, which diminish the water-holding capacity of the meat. Shrinkage during cooking causes the most noteworthy water loss at 60°C to 70°C, and it is assumed that water is removed by the pressure applied by the shrinking connective tissue on the aqueous solution in the extracellular void (Tornberg, 2005).

#### Protein

Raw breast meat with skin of the examined geese is characterized by protein content (17.2%) similar (17.55%) to that found by Damaziak et al. (2016) in the muscles of the same breed, although lower (20.5%) than that found by Oz and Celik (2015) in the muscles of Turkish geese. However, the protein content in raw muscles without skin (22.3%) is slightly higher than that found by Gumułka and Połtowicz (2020) in 10-wk-old geese of the same breed (21.21%).

In the present study, the protein content was higher (*P* ≤ 0.01) in geese muscles without skin than with skin (30.2 vs. 26.1 %). It has also been observed that heat processing impact (*P* ≤ 0.01) on the protein content in heat-treated muscles in comparison to raw muscles (19.7%). A significant (*P* ≤ 0.05) interaction has been also found between the type of muscle (with and without skin) and heat processing applied. The raw muscles with and without skin were characterized by significantly lower protein content than muscles subjected to heat processing (Table 2).

Belinsky and Kuhnlein (2000) found that heat processing has an impact on the protein content in breast muscles of Canada Goose: B>FR>OR (34.0>31.0>28.5 % respectively). Oz and Celik (2015) found a significant (*P* < 0.01) increase in the protein content in breast muscles of Turkish geese that were subjected to various kinds of heat processing (from 23.99 for grilled muscles to 32.17% for deep-fat-fried muscles). According to Yu et al. (2017), the types of heat processing bring about structural changes (denaturation of sarcoplasmic and myofibrillar protein) and molecular changes (protein carbonylation, modification of aromatic residues, creating the products of Maillard reaction) in meat protein.

Protein degradation into small peptides upon cooking has been seen to increase the protein digestibility in the colon without affecting the efficiency of small intestine digestion and without hindrance to meat protein residues in the colon (Bax et al., 2013).

#### Fat

The fat content of meat and heat processing are among the most important factors affecting the quality of meat before consumption. Muscles with skin had a higher percentage of fat than without (13.9 vs. 5.18%) (*P* ≤ 0.01). The fat content found in raw geese breast muscles with skin was lower (19.9%) than that reported by Damaziak et al. (2016) in the muscles of geese of the same breed (22.16%) but higher than the results achieved by Oz and Celik (2015) for Turkish geese (9.20%). This difference occurs because hybrid geese are bred for intensive meat production and thus grow faster and reach the inflection point earlier than traditional goose breeds. However, the fat content in raw skinless breast muscles is higher (3.3%) than reported by Gumułka and Połtowicz (2020) in 10-wk-old geese (2.23%) of the same breed. These differences could result from, for instance, feeding methods and the age of slaughtered animals.

Our research indicates a significant (*P* ≤ 0.01) interaction in fat content between the type of muscle (with and without skin) and heat processing. Raw muscles without skin contained (*P* ≤ 0.01 *or P* ≤ 0.05) less fat (3.3%) in comparison to muscles subjected to heat processing. The higher content of intramuscular fat resulted from the loss of water during heat processing. On the other hand, raw muscles with skin contained more fat (19.9%) than those subjected to heat processing. Fat loss simply results from liquefaction and consequent leakage. Similarly, Belinsky and Kuhnlein (2000) indicated that the type of heat processing impacts the fat content in the breast muscles with skin of Canada Goose: OR>B>FR (18.0>11.7>10.1% respectively). However, Oz and Celik (2015) did not find any impact of 7 types of heat processing on the fat content of breast muscles with skin of Turkish geese.

#### Ash

In this study, significant (*P* ≤ 0.01) changes in the ash content were found in muscles, regarding the presence of skin and the type of heat processing used. Muscles with skin contained less ash than those without skin (1.25 vs. 1.40%). Damaziak et al. (2016) found a similar content of ash (0.93%) in raw breast muscles of White Kołuda geese. However, the ash content in raw breast muscles without skin was slightly lower (1.23%) than that indicated by Gumułka and Połtowicz (2020) in 10-wk-old geese (1.31%).

The heat processing methods caused a significant general increase in ash content in muscles, in comparison to raw muscles (*P* ≤ 0.01). The highest ash content in muscles without skin has been found in grilled muscles (1.74%), while in grilled and fried muscles with skin, the content was 1.47 and 1.48% respectively. Also, Belinsky and Kuhnlein, (2000) indicated that heat processing affects the ash content in breast muscles with skin of Canada Goose: FR>OR>B (1.2>0.89>0.7% respectively).

Because the liquid phase of meat contains most of the minerals, the loss of these nutrients during heat treatment is significantly greater than for other ingredients. Simultaneously with the loss of water from muscle, there is a loss of soluble components, including collagen and sarcoplasmic proteins, with which minerals are associated. Retention of nutrients in food subjected to heat processing is important for dietary reasons. In our research on basic nutrients, the retention in breast muscles is between 28.3% and 103%. However, no significant difference in protein retention has been found, neither regarding the type of muscles (with or without skin), nor the type of heat processing. The lowest retention has been indicated for the fat in muscles with skin, and the highest for the skinless muscles WBC. When it comes to fat retention, the muscles without skin are characterized by a significantly *(P* ≤ 0.01) higher retention than those with skin (97.8 vs. 30.5%). A significant impact of heat processing is evident in ash retention. In muscles PF (81.9 %) and G (80.6%), there was higher retention of ash than in muscles WBC (68.4%).

Generally, cooking contributes to the loss of water-holding capacity, resulting in the concentration of proteins, fat, and ash in cooked meat (Jensen et al., 2014). Taking into account proteins, at temperatures up to 100°C, as in boiling in water, protein denaturation translates into effects such as enzymatic inactivation of lipases and proteases and improved digestibility. During baking, when the temperature is between 100 and 140°C, the digestibility of proteins is lowered by the formation of intramolecular and intermolecular covalent bonds. Similarly, during frying and grilling, where temperatures exceed 140°C, the destruction of amino acids, e.g., cysteine or tryptophan, with isomerization to configuration D takes place (Gómez et al., 2020). The thermal processing of meat products leads to fat melting. The released soluble fat escapes from the muscle at low temperatures. Fatty tissues are heat tolerant from 130°C to 180°C; however, some adipose cells may burst in the process. Higher temperatures and longer cooking times lead to greater lipid oxidation (Sánchez del Pulgar et al., 2012). In lipids, heat treatment brings about fats liquefaction. However, in case of triglyceride mixtures, it is difficult to establish their exact melting point, since before reaching the liquid state, they go through a pasty state, then smoky (at a different temperature depending on the type of fat), and finally decompose state. Therefore, heat treatment affects fat retention (Gómez et al., 2020)

### Mineral Composition

Many factors influence the mineral composition, such as species, breed, sex, age, muscle type, diet, and genetics, but also the cooking method (Alfaia et al., 2013). During the heat treatment, cooking losses due to mass transfer depend on not only the cooking conditions such as cooking method, cooking surface, cooking temperature and time but also the meat properties such as moisture, fat and protein content, pH value of the raw meat and the meat size (Gerber et al., 2009). Losses of minerals during heat processing of meat depending on the form in which they occur. Mineral components, which can be found in the form of soluble dissociated salts (part of Na, small amounts of P, Ca, and K, go to the leakage. Components, such as Fe, which combine with proteins, remain in the meat.

### Macroelements

Oat fattening gives unique health-promoting and taste qualities to White Kołuda geese meat and fat. Grazing as well as oat grains, which constitute the main feed for oat geese, are good sources of valuable fatty acids, tocopherols, and minerals.

The macroelement contents (P, Na, Ca, K, Mg) in raw goose breast muscles of geese and those subjected to heat processing are presented in Table 3. It has been found that raw muscles without skin or with skin contain the largest amount of potassium, and then also P, Na, Mg, and Ca. Similarly, Geldenhuys et al. (2015) indicated that the main macroelements in breast muscles of Egyptian geese are P, K, Mg, and Ca, irrespective of slaughter season and sex.

**Table 3:** Macroelements composition (mg/100 g DM) of raw and thermal treatment White Kołuda geese breast meat (MEAN, SEM).

Item	Meat	Raw	Thermal treatment					SEM	Level of significance		
			Water bath cooking (WBC)	Grilled (G)	Oven convection roasting (OCR)	Pan fried (PF)	Total				
									Meat (M)	Thermal treatment (MT)	M x MT
P	Without skin	910.9^A^	681.2^C^^b^	770.0^B^^a^	642.7^C^	794.6^B^	785.0^X^	19.1	0.000	0.001	0.001
	With skin	532.8^B^^b^	644.5^a^	695.0^A^	658.9^A^	723.8^A^	631.3^Y^	15.6			
	Total	721.7	662.9^B^	732.5^a^	650.8^B^^b^	759.2^A^	708.2	15.3			
	SEM	41.1	11.8	14.3	20.2	19.1					
Na	Without skin	256.5^A^	216.2^A^	226.7^A^	120.4^C^	158.6^B^	205.8^X^	9.38	0.001	0.001	0.001
	With skin	171.2^B^	156.9^B^	290.4^A^	121.1^C^^b^	144.3^C^^a^	175.9^Y^	10.3			
	Total	213.9^B^	186.5^B^	258.5^A^	120.8^C^^b^	151.4^C^^a^	190.8	7.14			
	SEM	10.5	11.4	15.9	2.83	4.08					
Ca	Without skin	10.5^B^	30.9^A^	20.2^a^	10.0^B^	8.3^B^^b^	15.1	1.62	0.657	0.001	0.002
	With skin	13.4^B^	17.3^B^	38.7^A^	10.6^B^	9.1^B^	17.1	2.05			
	Total	12.0^B^^b^	24.1^A^^a^	29.5^A^	10.3^B^	8.7^B^	16.1	1.30			
	SEM	0.65	3.15	4.45	0.45	0.85					
K	Without skin	1148.6^A^	443.8^D^	660.5^C^^b^	909.3^B^^a^	1062.7^B^	895.6^x^	48.0	0.027	0.001	0.001
	With skin	587.2^b^	570.9^b^	489.0^B^	710.0	1047.3^A^^a^	815.3^y^	178.8			
	Total	867.9^b^	507.4^C^	574.8^C^^c^	809.6^B^^b^	1505.0^A^^a^	855.4	92.0			
	SEM	68.4	32.7	54.9	35.1	496.3					
Mg	Without skin	119.6^A^^a^	104.6^A^	110.4^A^	82.9^B^^c^	99.4^b^	106.1^X^	2.70	0.001	0.001	0.001
	With skin	78.5^B^	93.5^B^	123.1^A^	85.5^B^	89.7^B^	91.5^Y^	3.08			
	Total	99.1^B^^C^^a^	99.1^B^	116.7^A^	84.2^C^^b^	94.6^BC^	98.8	2.21			
	SEM	4.93	3.05	4.60	1.32	2.25					

Means within a row followed by different superscript letters differ significantly.

A,B,C,D *P* ≤ 0.01.

a,b *P* ≤ 0.05. Means within a row followed by different superscript letters differ significantly.

X,Y *P* ≤ 0.01.

x,y *P* ≤ 0.05. Means within a column followed by different superscript letters differ significantly. (n = 6 raw breast muscles with skin and n = 12 without skin; n = 6 breast muscles with skin and n = 6 without skin for each kind of heat treatment).

In our research, the contents of P, Na, Ca, K and Mg in raw breast muscles without skin were higher (910.9; 256.5; 10.5; 1148.6 119.6 mg/100 g DM respectively) than the results published in 2020 for the raw meat of breast without skin from domesticated geese (312;0 87;0 13;0 420;0 24.0 mg/100 g DM respectively), and Canadian geese (256.0; 50.0; 4.0; 336.0; 29.0 mg/100 g DM respectively). Moreover, the contents of P, Na, Ca, K and Mg that our research indicates in the raw breast muscles with skin were higher (532.8; 171.2; 13.4; 587.2 78.5 mg/100 g DM respectively) than that published in 2020 for the raw meat of breast without skin from domesticated geese (234.0; 73.0; 12.0; 308.0; 18.0 mg/100 g DM respectively).

The present study showed that the general content of P, Na, Mg (*P* ≤ 0.01), and K (*P* ≤ 0.05) were higher in the muscles without skin than in the muscles with skin. In the case of Ca content, such significance has not been found. For all macroelements, a significant (*P* ≤ 0.01) interaction was found between a type of meat and heat processing used (Table 3).

#### Phosphorus

The content of P in raw breast meat of geese with skin was found to be similar to that indicated by Oz and Celik (2015) for Turkish geese (532.8 vs. 558.0 mg/g DM). The highest content of P was found in raw muscles without skin (910.9 mg/100 DM) (*P* ≤ 0.01), in comparison to those heat processed. The lowest amount has been found in the skinless muscle OCR and muscle with skin WBC. According to 2020, the P content in the carcass meat of domestic goose, both with and without skin, roasted, was lower (309.0 and 270.0 mg/100 g DM respectively) than that noted in our study for the muscles OCR (642.7 and 658.9 mg/100 g DM respectively). Likewise, Geldenhuys et al. (2013) found a lower content of P (195.5 mg/100 g DM) in the breast meat of Egyptian geese cooked in preheated (160°C) conventional ovens. On the other hand, Oz and Celik (2015) have found no significant differences in P contents between the breast muscles of Turkish geese, with skin, subjected to 7 different types of heat processing.

#### Sodium

Taking into account the prevention of cardiovascular diseases, there is a high demand among the consumers for foods with low sodium content, and in the case of dietary therapy for arterial hypertension, a low-sodium diet is advised. The Na content in geese's raw breast muscles with skin analyzed in the present study was lower by half than the content found by Oz and Celik (2015) in Turkish geese (171.2 vs. 351.0 mg/g DM). However, the cited authors salted the examined carcasses after slaughter.

During an analysis of heat processing applied, it has been found that the muscles OCR without or with skin (120.4 and 121.1 mg/100 DM respectively) showed the lowest (*P* ≤ 0.01) content of Na while the highest content has been found in G (290.4 and 258.5 mg/100 g DM respectively).

According to 2020, the content of Na in the carcass meat of domestic goose, without or with skin, subjected to roasting was lower (76.0 and 70.0 mg/100 g DM respectively) than that found in our research in the breast muscles, without or with skin, OCR (120.4 and 121.1 mg/100 g DM respectively). Also, other authors (Geldenhuys et al., 2013) indicated a smaller (22.0 mg/100 g DM) content of Na in the breast muscles of Egyptian geese cooked in preheated (160°C) conventional ovens. However, Oz and Celik (2015) have found no significant differences in Na contents of breast muscles of Turkish geese, with skin, subjected to 7 different types of heat processing. Na is the major cation in extracellular fluids, therefore, the loss of this nutrient along with thermal leakage during cooking is significantly higher than intracellular ions and minerals bound to proteins.

#### Calcium

The research showed that the Ca content in raw breast muscles with skin was much lower than that indicated by Oz and Celik (2015) in breast muscles of Turkish geese (13.4 vs. 168.0 mg/100 g DM). The highest content of Ca (*P* ≤ 0.01) was indicated in the muscles without skin WBC (30.9 mg/100 g DM) and G with skin (38.7 mg/100 DM). However, one of the reported disadvantages of grilling is the risk of the formation of HAAs (Heterocyclic Aromatic Amines), as these compounds are formed when meat is processed at a temperature above 200°C. Grilled meat is also prone to the formation of PAHs (Polycyclic Aromatic Hydrocarbons) and it has been reported that following an increase in cooking time, a rise in benzo (a) pyrene (B(a)P) content will occur (Gibis, 2016; Sahin et al., 2020). Other authors (Belinsky and Kuhnlein, 2000) have observed changes in the Ca content affected by heat processing of breast muscles with skin of Canada Goose: OR>FR>B (7.44>6.53>5.83 mg/100 g respectively). According to 2020, the content of Ca in the carcass meat of domestic goose without or with skin, roasted, was higher (14.0 and 13.0 mg/100 g DM respectively) than that indicated by our research for muscles (10.0 and 10.6 mg/100 g DM respectively). Also, Geldenhuys et al. (2013) have noted lower (12.3 mg/100 g DM) content of Ca in breast muscles of Egyptian geese cooked in preheated conventional ovens. However, no significant changes in Ca content in breast muscles with skin of Turkish geese, subjected to various form of heat processing, was found by Oz and Celik (2015).

#### Potassium

In our research, we found that the raw muscles without skin had (*P* ≤ 0.01) the highest K content (1,148.6 mg/100 g DM). The K content in raw breast muscles with skin was higher than that noted by Oz and Celik (2015) in breast muscles of Turkish geese (587.2 vs. 61.2 mg/g DM).

Regarding heat processing, the WBC and G muscles with skin were characterized by the lowest content of K (443.8 and 489.0 mg/100 g DM respectively). Similarly, Oz and Celik (2015) concluded that in terms of cooking, cooking methods had a significant effect (*P* < 0.05) on the K content of breast meat. The highest content of K was indicated in breast muscle with skin, pan fried without fat or oil (121.0 mg/100 g DM), whereas the lowest content was in muscles that were boiled (52.5 mg/100 g DM). Nevertheless, according to 2020, the content of K in the carcass meat of domestic goose, without or with skin, roasted, was lower (388.0 and 329.0 mg/100 g DM respectively) than the contents found in our study of samples OCR (909.3 and 710.0 mg/100 g DM respectively). Likewise, Geldenhuys et al. (2013) have indicated a lower (180.1 mg/100 g DM) content of K in breast muscles of Egyptian geese cooked in preheated conventional ovens.

#### Magnesium

The content of Mg in raw breast muscles with skin was slightly higher than that indicated by Oz and Celik (2015) in the breast muscles of Turkish geese (78.5 vs. 61.1 mg/g DM). The type of heat treatment applied reduced the Mg content in skinless muscles and increased in muscles with skin (*P* ≤ 0.01). The lowest content of Mg has been found in OCR skinned muscles (82.9 mg/100 g DM), and the highest in the G muscles with skin (123.1 mg/100 g DM) (*P* ≤ 0.01). The content of Mg in the roasted meat of domestic goose (2020), without or with skin, was lower (25.0 and 22.0 mg/100 g DM respectively) than that indicated in our research in breast muscles OCR (82.9 and 85.5 mg/100 g DM respectively). Similarly, Geldenhuys et al. (2013) have found a lower (32.5 mg/100 g DM) content of Mg in breast muscles of Egyptian geese cooked in preheated conventional ovens. However, Oz and Celik (2015) have not found any significant changes in Mg content in the breast muscles of Turkish geese subjected to various types of heat processing.

Regarding macroelements, a significant (*P* ≤ 0.01) influence of the type of muscle (with or without skin) and type of heat processing on retention of Na, Ca, K, and Mg has been found (Table 3). Retention of these elements, depending on the factors applied, is between 24.6 and 188.2%. In skinless muscles WBC the highest retention has been found for Ca (188.2%), and the lowest for K (24.6%). A significant (*P* ≤ 0.05) interaction has been found only for the retention of Na. Regarding the type of heat processing, the lowest retention of Na has been found in OCR muscles without or with skin (28.7 vs. 36.8%), while the highest was found in G muscles with skin (87.1%) and WBC (53.5%) without skin. The retention of K was significant (*P* ≤ 0.05) only for the muscles without skin, and the highest retention has been found in muscles PF (58.7%), and the lowest in samples WBC (24.6%). On the other hand, the highest retention of Mg has been found in G muscles without skin (93.5%), and the lowest in samples OCR (65.6%). The retention of P, depending on heat processing and the presence of skin, was not statistically significant (Table 4).

**Table 4:** Retention coefficients macroelements in the White Kołuda geese breast meat after thermal treatment (MEAN, SEM).

Item	Meat	Raw	Thermal treatment					SEM	Level of significance		
			Water bath cooking (WBC)	Grilled (G)	Oven convection roasting (OCR)	Pan fried (PF)	Total				
									Meat (M)	Thermal treatment (MT)	M x MT
P	Without skin	-	47.2	49.5	43.2^b^	56.6^a^	49.1	1.81	0.705	0.864	0.537
	With skin	-	68.0	65.7	59.0	55.6	62.1	7.09			
	Total	-	57.6	57.6	51.1	56.1	55.6	3.83			
	SEM	-	7.63	6.84	5.23	11.5					
Na	Without skin	-	53.5^A^	52.2^A^	28.7^B^	39.3	43.4^Y^	3.47	0.001	0.001	0.046
	With skin	-	52.7^B^	87.1^A^	36.8^B^	47.0^B^	55.9^X^	6.09			
	Total	-	53.1^A^^B^	69.7^A^	32.8^C^	43.2^B^	49.7	3.67			
	SEM	-	3.24	8.80	2.60	2.22					
Ca	Without skin	-	188.2^A^	144.1^A^^B^^a^	59.8^B^^b^	51.1^B^	110.8^x^	20.1	0.032	0.001	0.228
	With skin	-	91.9	151.7^A^	41.1^B^	43.4^B^	82.0^y^	16.6			
	Total	-	140.0^A^	147.9^A^	50.5^B^	47.2^B^	96.4	13.1			
	SEM	-	26.8	23.73	6.90	3.83					
K	Without skin	-	24.6^B^	33.8^BC^^b^	47.9^A^^C^^a^	58.7^A^	41.3^y^	4.08	0.038	0.041	0.528
	With skin	-	65.8	51.3	68.2	98.9	71.0^x^	11.7			
	Total	-	45.2	42.6	58.1	78.8	56.1	6.80			
	SEM	-	16.7	14.5	8.08	14.0					
Mg	Without skin	-	55.1	54.6	42.1	52.6	51.1^Y^	2.36	0.001	0.040	0.701
	With skin	-	80.7	93.5^a^	65.6^b^	73.3	78.3^X^	5.09			
	Total	-	67.9	74.0	53.8	63.0	64.7	3.95			
	SEM	-	8.28	10.2	6.40	5.40					

Means within a row followed by different superscript letters differ significantly.

A,B,C,D *P* ≤ 0.01.

a,b *P* ≤ 0.05. Means within a row followed by different superscript letters differ significantly.

X,Y *P* ≤ 0.01.

x,y *P* ≤ 0.05. Means within a column followed by different superscript letters differ significantly. (n = 6 breast muscles with skin and n = 6 without skin for each kind of heat treatment).

### Microelements

The content of microelements (Fe, Zn, Cu, and Mn) in raw breast muscles of geese and those subjected to heat processing is presented in Table 5. It has been found that the raw muscles with or without skin contain most Fe, and subsequently, Zn, Cu, and Mn. Likewise, Geldenhuys et al. (2015) indicated that the main microelements in breast muscles of Egyptian geese are Fe, Zn, Cu, and Mn, regardless of slaughter season and sex.

**Table 5:** Microelements composition (mg/100 g DM**)** of raw and thermal treatment White Kołuda geese breast meat (MEAN, SEM).

Item	Meat	Raw	Thermal treatment					SEM	Level of significance		
			Water bath cooking (WBC)	Grilled (G)	Oven convection roasting (OCR)	Pan fried (PF)	Total				
									Meat (M)	Thermal treatment (MT)	M x MT
Fe	Without skin	14.2^B^	20.3^A^	17.5^A^^a^	15.2^A^^b^	15.2^A^^b^	16.1^X^	0.41	0.001	0.001	0.001
	With skin	7.2^B^	17.0^A^	17.6^A^	13.9^A^	13.5^A^	13.0^Y^	0.68			
	Total	11.1^B^	18.7^A^^a^	17.5^A^	14.6^A^^b^	14.3^A^^b^	14.6	0.44			
	SEM	0.69	0.70	0.48	0.24	0.35					
Zn	Without skin	4.91	4.54	4.61	4.82	4.55	4.71^X^	0.09	0.001	0.017	0.001
	With skin	3.35^B^	3.61	4.78^A^^a^	3.70^b^	3.88^b^	3.78^Y^	0.12			
	Total	4.13^b^	4.07^b^	4.70^a^	4.26	4.16	4.24	0.09			
	SEM	0.22	0.19	0.09	0.21	0.13					
Cu	Without skin	1.84^A^	1.62^A^	1.46	1.16^B^	1.13^B^	1.51^X^	0.06	0.001	0.001	0.046
	With skin	1.22^A^	1.13^A^	1.58^A^	0.85^B^	0.87^B^	1.14^Y^	0.05			
	Total	1.53^A^	1.38^A^	1.52^A^	1.01^B^	1.00^B^	1.33	0.05			
	SEM	0.08	0.09	0.07	0.07	0.05					
Mn	Without skin	0.17	0.27^a^	0.20	0.13	0.11^b^	0.18	0.02	0.840	0.001	0.001
	With skin	0.15^B^	0.14^B^	0.38^A^	0.11^B^	0.13^B^	0.18	0.02			
	Total	0.16^B^	0.20	0.29^A^	0.12^B^	0.12^B^	0.18	0.01			
	SEM	0.02	0.04	0.04	0.01	0.02					

Means within a row followed by different superscript letters differ significantly.

A,B,C,D *P* ≤ 0.01.

a,b *P* ≤ 0.05. Means within a row followed by different superscript letters differ significantly.

X,Y *P* ≤ 0.01. Means within a column followed by different superscript letters differ significantly. (n = 12 raw breast muscles with skin and n = 12 without skin; n = 6 breast muscles with skin and n = 6 without skin for each kind of heat treatment).

In the present research, the contents of Fe, Zn, Cu, and Mn in raw breast muscles without skin (14.2; 4.91; 1.84; 0.17 mg/100 g DM respectively) were higher than those published in 2020 for the breast meat of domesticated geese (2.57; 2.34; 0.306; 0.024 mg/100 g DM respectively) and Canadian geese (5.91; 1.68; 0.443; 0.050 mg/100 g DM respectively). Our research indicated that the contents of Fe, Zn, Cu, and Mn in raw breast muscles with skin were higher (7.2; 3.35; 1.22; 0.15 mg/100 g DM respectively) than that found by 2020 for raw meat with skin of domesticated goose (2.5; 1.72; 0.27; 0.02 mg/100 g DM respectively).

Analyzed muscles with skin contained more Fe, Zn, and Cu than those without skin (*P* ≤ 0.01). It was not found the same correspondence when it comes to Mn. A significant (*P* ≤ 0.01) impact of heat processing on the contents of microelements in muscles was found (Table 5).

#### Iron

Iron present in meat impacts its storage value because iron released from heme during heat processing (clefting of the porphyrin ring) and transformed into nonheme Fe is one of the catalyzers of lipid oxidation. At the same time, changes in the proportions of heme and nonheme Fe may affect its bioavailability from meat as a component of a diet (Lombardi-Boccia et al., 2002).

Analyzed raw muscles without and with skin were characterized by the lowest content of Fe (14.2 and 7.2 mg/100 g DM respectively), but these are much higher values than those indicated by Oz and Celik (2015) in breast muscles of Turkish geese (7.2 vs. 1.01 mg/100 g DM).

Heat processing significantly impacted the concentration of Fe in muscles. It could be related to larger water losses that take place during heat processing, and used temperature >55°C leads to quick denaturation of myoglobin that allows releasing iron from heme (Kristensen and Purslow, 2001). Similarly, Belinsky and Kuhnlein (2000) have found that heat processing affects the Fe content in breast muscles with skin of Canada Goose: B>FR>OR (8.98>7.81>6.78 g/100 g respectively). Moreover, Oz and Celik (2015) indicate a significant (*P* ≤ 0.05) increase in Fe content in beast muscles with skin of Turkish geese, pan fried without and/or oil (2.5 mg/100 g DM), in comparison to raw muscles (1.01 mg/100 g DM). Additionally, muscles subjected to other types of heat processing indicate a larger Fe content in comparison to raw muscles. However, as data collected by 2020 show, the content of Fe in the roasted meat of domestic goose without and with skin is lower (2.87 and 2.83 mg/100 g DM respectively) than that indicated in our study in muscles OCR (15.2 and 13.9 mg/100 g DM respectively). Geldenhuys et al. (2013) also found lower (7.5 mg/100 g DM) content of Fe in breast muscles of Egyptian geese, cooked in preheated conventional ovens, although they are wild game, whose breast muscle comprises mainly red, rapidly shrinking, oxidative glycolytic fibers (type IIa), with a small percentage of glycolytic fibers (type IIb).

#### Zinc

The content of Zn in examined raw breast muscles of geese with skin is much higher than that indicated by Oz and Celik (2015) in the breast muscles of Turkish geese (3.35 vs. 1.5 mg/g DM). Regarding Zn, we have found that a significant impact of heat processing is only evident in muscles with skin, and its highest value is noted in grilled muscles (4.78 mg/100 g DM). Other authors (Belinsky and Kuhnlein, 2000) have observed an impact of heat processing on Zn content in breast muscles with skin of Canada Goose: B>OR>FR (3.25>3.05>2.90 g/100 g DM respectively). On the other hand, according to 2020, the Zn content in the roasted meat of a domestic goose without or with skin was lower (3.72 and 2.62 mg/100 g DM respectively) than that indicated in our research in breast muscles OCR (4.82 and 3.70 mg/100 g DM respectively). Similarly, Geldenhuys et al. (2013) have found a lower (2.1 mg/100 g DM) content of Zn in breast muscles of Egyptian geese cooked in preheated conventional ovens. However, Oz and Celik (2015) have indicated no changes to Zn content in breast muscles with skin of Turkish geese, subjected to 7 different types of heat processing.

#### Copper

The content of Cu indicated in the examined raw breast muscles with skin was much higher than the value found by Oz and Celik (2015) in the breast muscles of Turkish geese (1.22 vs. 0.15 mg/g DM). The highest content of Cu (1.84 mg/100 g DM) (*P* ≤ 0.01) has been found in the muscles without skin, and all 4 types of heat processing decrease its concentration. On the other hand, the highest content of Cu has been indicated in the grilled muscles with skin (1.58 mg/100 g DM). Belinsky and Kuhnlein (2000) noted that heat processing affects the content of Cu in the breast muscles with skin of Canada Goose: B>FR>OR (0.50>0.45>0.40 mg/100 g DM respectively). According to 2020, the content of Cu in the roasted meat of domestic goose, without or with skin, was lower (0.276 and 0.264 mg/100 g DM respectively) than that noted in the present study in OCR breast muscles (1.16 and 0.85 mg/100 g DM respectively). Similarly, Geldenhuys et al. (2013) indicated a lower (0.5 mg/100 g DM) content of Cu in breast muscles of Egyptian geese cooked in preheated conventional ovens. However, Oz and Celik (2015) have not noted any significant changes in Cu content in breast muscles with skin of Turkish geese that were subjected to various types of heat processing.

#### Manganese

Oz and Celik (2015) found a much smaller content of Mn in breast muscles of Turkish geese than we have indicated in our results (0.15 vs. 0.02 mg/100 g DM) for raw breast muscles with skin. In the present study, skinless WBC muscles, unlike the PF muscles, had the highest Mn content (0.25 vs. 0.11 mg/100 g DM). However, in muscles with skin, the highest content of Mn has been found in grilled muscles (0.38 mg/100 g DM) (*P* ≤ 0.01*)* as contrasted with other methods of heat processing. As indicated in 2020, the content of Mn in the roasted meat of domestic goose, without and with skin, was lower (0.024 and 0.023 mg/100 g DM respectively) than that found in our study for the breast muscles OCR (0.13 and 0.11 mg/100 g DM respectively). Likewise, Geldenhuys et al. (2013) have noted lower (0.1 mg/100 g DM) content of Mn in breast muscles of Egyptian geese cooked in preheated conventional ovens. However, Oz and Celik (2015) have indicated no significant changes in Mn content in breast muscles with skin of Turkish geese subjected to various types of heat processing.

In terms of all microelements, the interaction between the type of breast muscle (with or without skin) and the heat processing method is statistically significant (Table 6). Regarding microelements, it has been found that there is a significant (*P* ≤ 0.01) impact of the presence of the skin on the meat on the retention of Fe and Zn, while the method of heat processing impacts the retention of Fe and Cu. The retention of microelements, depending on applied factors, is between 36.4 and 123.5%. Nevertheless, an interaction in microelements retention between breast muscles (with or without skin) and heat processing was not statistically significant. However, the highest (123.5%) (*P* ≤ *0.*01) retention of Fe is indicated in the WBC muscles with skin. When it comes to Zn, its retention was not impacted by the method of heat processing in any significant way. The methods of heat processing decreased the retention of Cu in skinned muscles, and its highest retention was found in WBC muscles (58.2%) (*P* ≤ 0.05). No significant changes in retention of Mn are noted, irrespective of the type of muscle (with or without skin) or type of heat processing.

**Table 6:** Retention coefficients microelements in the White Kołuda geese breast meat after thermal treatment (MEAN, SEM).

Item	Meat	Raw	Thermal treatment					SEM	Level of significance		
			Water bath cooking (WBC)	Grilled (G)	Oven convection roasting (OCR)	Pan fried (PF)	Total				
									Meat (M)	Thermal treatment (MT)	M x MT
Fe	Without skin	-	90.4^a^	72.1	64.9^b^	67.5^b^	73.7^Y^	3.63	0.001	0.001	0.466
	With skin	-	123.5^A^	114.6^a^	91.1^B^^b^	95.2^B^	106.1^X^	4.43			
	Total	-	107.0^A^^a^	93.4^b^	78.0^B^^c^	81.3^B^	89.9	4.39			
	SEM	-	8.32	9.68	6.28	6.90					
Zn	Without skin	-	62.9	54.6	63.2	55.0	58.9^Y^	2.19	0.008	0.951	0.051
	With skin	-	64.4	79.4	61.8	72.9	69.6^X^	3.29			
	Total	-	63.6	67.0	62.5	64.0	64.3	2.23			
	SEM	-	1.82	7.57	2.50	4.73					
Cu	Without skin	-	58.2^a^	48.7	38.9^b^	40.3^b^	46.6	3.49	0.593	0.004	0.276
	With skin	-	53.8	67.1	36.4	40.2	49.4	4.40			
	Total	-	56.0^a^	57.9^A^^a^	37.6^b^	40.3^B^	48.0	2.76			
	SEM	-	5.22	6.11	1.03	2.92					
Mn	Without skin	-	106.8	77.0	47.0	43.6	68.6	14.6	0.445	0.056	0.225
	With skin	-	62.3	158.6	43.5	60.1	81.1	17.1			
	Total	-	84.6	117.8	45.3	51.8	74.9	11.0			
	SEM	-	23.37	30.89	2.76	9.11					

Means within a row followed by different superscript letters differ significantly.

A,B,C,D *P*≤0.01.

a,b *P* ≤ 0.05. Means within a row followed by different superscript letters differ significantly.

X,Y *P* ≤ 0.01. Means within a column followed by different superscript letters differ significantly. (n = 6 breast muscles with skin and n = 6 without skin for each kind of heat treatment).

Similarly, boiling of pork loin increases the mineral content as the consequence of water lost during cooking (Tomović et al., 2015). During the boiling process, inorganic materials such as phosphorus and calcium in the food are easy to lose with water. There is general agreement that zinc, copper, and iron are the most stable minerals in cooked meats and the degree of meat shrinkage during cooking affects significantly the retention of minerals. On the other hand, the greater retention of ash in grilled and fried meat results from the specificity of processing. The grilling and pan frying methods, applied in this study, did not require water, which probably allowed for higher retention of the minerals. Frying has been proven to increase the surface temperature of meat quickly to 115°C to 120°C or above 120°C. In such temperature proteins form a solid film on the surface of the meat so the solubles, like nitrogen and inorganic salts, are lost to a lesser extent (Yong et al., 2019). Due to high-heat air during roasting, the raw meat forms a hard shell on the surface, protecting from loss of internal leachate. In addition, divalent minerals are better retained during processing than monovalent minerals, which may be due to their stronger relationship with proteins (Gerber et al., 2009).

It has been demonstrated that in the meat with skin increased (not always significantly) the retention of protein, ash, Na, K, Fe, Zn, Cu, and Mn. Skin provided a kind of protection (barrier) for the muscles against water loss during cooking, hot air during baking, hot plates grilling, and oil frying. The results of this study are relevant to the consumer because of the Nutrient Reference Values (NRVs). NRVs are a set of values used in nutrition labeling derived from authoritative recommendations for daily nutrient intake. These recommendations are based on the best available scientific knowledge of the daily amount of energy or nutrient needed for good health. NRVs do not appear on the label but they are used in nutrition labeling to show the contribution to healthy nutrient intake a portion of food (Lewis, 2019). Taking the above into account, from a dietary point of view, the most advantageous were the OCR muscles without skin due to the lowest energy value, as well as content and retention of fat, P, and Na. However, taking into account the contents of other minerals and their retention, the most optimal form of heat processing for meat without skin was water bath cooking. 100 g of WBC treated meat without skin may meet the Nutrient Reference Values-Requirements (NRVs-R) of an adult person for Ca, Mg, Fe, Cu, and Mn in 3.1; 33.7; 145; 180 and 9%. In turn, for meat with skin, the most optimal form of heat processing in terms of retaining minerals was grilling. 100 g of such meat may meet the NRVs-R requirements of an adult person for Ca, Mg, Fe, Cu, and Mn in 3.9; 39.7; 125.7; 175;6 and 12.7%. Therefore, goose breast muscles, as analyzed here, cannot be considered as a source of calcium, since it provides less than 4% of NRVs-R (Table 7).

**Table 7:** Nutrient reference values-requirements (NRVs-R) per 100 g and their implementation in the breast muscles of geese subjected to water bath cooking (WBC) and grilling (G).

Minerals	NRVs-R (mg/100 g)	WBC muscles without skin (mg/100 g DM)	% NRV/100 g	G muscles with skin (mg/100 g DM)	% NRV/100 g
P	700	681.2	97.3	695.0	99.3
Ca	1000	30.9	3.1	38.7	3.9
Mg	310	104.6	33.7	123.1	39.7
Fe	14	20.3	145.0	17.6	125.7
Zn	11	4.54	41.3	4.78	43.5
Cu	0.9	1.62	180.0	1.58	175.6
Mn	3	0.27	9.0	0.38	12.7

Since available literature contains few works on the influence of heat processing on the content of examined mineral elements in goose breast muscles (with or without skin), a wider discussion of achieved results is not possible.

## CONCLUSION

The application of various heat processing methods (WBC, OCR, G, and PF) had a significant impact on the energy and nutritious value of goose meat (with and without skin). The results of our study may be useful for consumers in making dietary choices. Breast muscles of examined geese, depending on the presence of skin and type of heat processing, provide consumers with nutrients, including mineral elements, and thus may be a valuable component of a varied diet.

## DISCLOSURES

The authors declare no conflicts of interest in publication.
